# Effects of Manual Therapy on Fatigue, Pain, and Psychological Aspects in Women with Fibromyalgia

**DOI:** 10.3390/ijerph17124611

**Published:** 2020-06-26

**Authors:** Yolanda Nadal-Nicolás, Jacobo Ángel Rubio-Arias, María Martínez-Olcina, Cristina Reche-García, María Hernández-García, Alejandro Martínez-Rodríguez

**Affiliations:** 1 Faculty of Medicine, Miguel Hernández University of Elche, 03202 Elche, Spain; yolanda.nadal@umh.es; 2 Department of Health and Human Performance, Faculty of Physical Activity and Sport Science, Polytechnic University of Madrid, 28040 Madrid, Spain; ja.rubio@upm.es; 3 Faculty of Health Sciences, University of Alicante, 03690 Alicante, Spain; mmo36@alu.ua.es (M.M.-O.); mhg30@alu.ua.es (M.H.-G.); 4 Faculty of Nursing, Catholic University of Murcia, 30107 Murcia, Spain; creche@ucam.edu; 5 Faculty of Sciences, Department of Analytical Chemistry, Nutrition and Food Sciences, University of Alicante, 03690 Alicante, Spain; 6 Alicante Institute for Health and Biomedical Research (ISABIAL), 03010 Alicante, Spain

**Keywords:** chronic disease, muscle fatigue, chronic pain, sleep disorders

## Abstract

Fibromyalgia is a condition characterised by chronic widespread muscle pain and fatigue, sleep disturbances, cognitive disorders, and mood disturbance. The purpose of this study was to determine the effectiveness of a manual therapy technique performed with moderate digital pressure in women with fibromyalgia (*n* = 24). In this randomised, controlled trial, the participants were randomly assigned to the experimental group or placebo group. The experimental group was assisted with manual therapy sessions based on connective tissue massage, whereas the placebo group was “treated” with ultrasound sessions performed without conductive gel and with the machine turned off as the placebo. Fatigue severity scale (FSS), visual analogical scale (VAS), Pittsburgh sleep quality index (PSQI), and profile of mood states (POMS-29) were completed before and after the intervention. In the experimental group (manual therapy), significant results were obtained on a VAS scale, referring to the neck pain in patients with fibromyalgia (*p* < 0.001). Correlations showed a relationship between fatigue and sleep variables (*R* = 0.411; *p* = 0.046) and pain variables with the POMS anger-hostility subscale (*R* = 0.436; *p* = 0.033). Although the size of the sample could be a limitation, the study concluded that the application of manual therapy in fibromyalgia patients performed with moderate pressure for 15 min on the posterior cervical musculature decreased the perception of pain, muscle fatigue, and the state of tension-anxiety.

## 1. Introduction

Fibromyalgia (FM) is a chronic musculoskeletal disease of unknown aetiology and is characterised by pain diffused throughout the body and hyperalgesia. Patients with FM also have functional and emotional disorders, including persistent fatigue, sleep disturbances, paresthesia, cognitive disorders, and mood disturbance [1]. Until 2016, the diagnostic criteria for fibromyalgia included the assessment of pain at 19 sites and a 4-item symptom severity scale from which an overall fibromyalgia severity score, the polysymptomatic distress (PSD) scale, could be calculated [2]. In 2016, a modification added a widespread pain criterion and clarified scoring (2016 criteria) [3]. The new criteria for diagnosis combines the concept of generalised chronic pain (such as a generalised pain index covering 19 regions) with the presence of an additional measure for fatigue, sleep, cognitive symptoms, mood symptoms, and other sources of pain. The symptom severity score and the combination of the generalised pain index provides a maximum score of 31. For diagnosis, patients should score a minimum of 3 or higher on the generalised pain index, with a total score of 12 when combined with the symptom severity score [3]. Studies have found prevalence lower than 1% in Denmark, 2% in Spain, and an estimated 2% to 3.3% in North America [4]. Generally, it is believed that FM affects between 2% and 4% of the world’s population, and the prevalence is higher among women aged between 50 to 80 years, reaching 7% [5].

In the absence of definitive pathology, the critical aspect for evaluating FM patients relies upon patients to report the presence and degree of symptoms [6]. Because of the symptoms, it has been observed [7,8] that FM can affect daily functioning, both physically and psychologically.

While fatigue is a common complaint for individuals with fibromyalgia, pain is the defining characteristic for diagnosing it [9]. One of the most challenging aspects of fibromyalgia is the variable nature of pain. It can be associated with morning stiffness as well as increasing pain throughout the day [6,10]. Patients with fibromyalgia exhibit dysregulated functioning of the hypothalamus–pituitary–adrenal–cortex axis [11] and central sensitisation [12], which leads to increased pain sensitivity. Pain in individuals with fibromyalgia has been associated with greater disease severity, reduced function, and symptoms of fibromyalgia [13,14]. Thus, pain is a significant symptom that may affect physical functioning.

Poor sleep is reported by almost 80% of patients with fibromyalgia [11]. Epidemiological studies indicate that lower sleep quality is a risk factor for fibromyalgia; poor sleep is strongly and dose-dependently associated with symptom severity [14,15] in the fibromyalgia population. As part of the American College of Rheumatology (ACR) 2010 diagnostic criteria for fibromyalgia, “waking unrefreshed” is one of the symptoms [11]. There are interactions between sleep disorders, neuroendocrine and immune disorders, and clinical symptoms present in FM. Therefore, sleep disturbances can be both a cause and a consequence of FM [16]. Poor sleep quality and pain can have a significant impact on the overall quality of life of FM patients [11]. It has been seen that quality of sleep can be an important mediator of the relationship between pain, distress, emotional functioning [14], and anxiety [17]. Some authors point out that pain by itself does not directly produce emotional distress [14].

Anxiety, which is a key symptom in fibromyalgia, is associated with higher levels of pain and neuropsychological disorders in these patients [18]. It is also associated with higher fibromyalgia impact, and patients with high levels of anxiety usually present an increased risk of severe fibromyalgia. Some research suggests that the prevalence of depressive and anxiety disorders in FM patients is significantly higher compared to the prevalence in the general population; this prevalence is 20–80% and 13–63.8% of cases, respectively [19].

Severe anxiety and/or depression can impede the ability to comply with nonpharmacological therapy, which is why interactions with chronic pain and fatigue can become cyclical and self-perpetuating. In FM, it is known that a negative mood can lead to a poor perception of physical health [20]. Depression can increase the perception of pain, producing a vicious cycle of depression/pain/depression [21].

The complex symptomatology of FM mainly involves three areas: aspects of physical health (musculoskeletal system), pain regulation mechanisms (neuroendocrine system), and factors related to psychological well-being and mental health [22]. Despite the fact that in recent years some studies have explored the complex relationships between the different aspects and symptoms of the disease [14,23], it is still necessary to deepen both the explanatory mechanisms that affect its severity and the most appropriate treatments to minimize its main symptoms. According to clinical practice guidelines, there are various forms of treatment for FM, from patient education, drug treatment, physical therapy, and psychology, to some alternative therapies such as yoga, taichi, or acupuncture [24]. In most patients, a multidisciplinary approach, combining nonpharmacological and pharmacological treatments, is needed [17].

Randomised controlled trials have shown that multiple nonpharmacological treatments such as psychotherapy, exercise therapy, education, and physiotherapy are effective in the reduction of FM symptoms [24]. The main causes for patients to seek medical care are musculoskeletal pain and sleep disorders [25]. Patients often seek relief from their symptoms [26]. Physical exercise treatment has been shown to show promising results for this population [27].

Manual therapy has been defined in different ways, one of them as the manipulation of soft tissues and joints using the hands and another as the systematic mapping of soft tissue with rhythmic pressure to prevent, develop, maintain, rehabilitate, or increase physical function or relieve pain [28]. In physiotherapeutic practise, manual therapy plays an important role in the treatment of patients with musculoskeletal disorders. Chronic back pain, migraines, anxiety, hypertension, depression, and many other physical and psychological conditions have been shown to respond positively to manual therapy [29]. Connective tissue massage is considered an important element of manual therapy, dealing with the skin and subcutaneous tissue. However, most of the literature reports the beneficial effects of manual therapy on healthy people and there are very few studies that report these effects in FM patients.

Despite this, Cimmino et al. has observed that massage is the therapeutic modality used by 75% of patients with fibromyalgia [30]. However, there is only moderate evidence (level B) to recommend this therapy for FM patients because the massage can be extremely painful; however, many of the patients prefer it because the benefits reward them later. According to Roberts [29], the intensity of the massage should be moderate to save excessive pain and be beneficial.

The application of different types of massage in FM patients, including connective tissue massage, has benefits in terms of improving FM symptoms, especially pain, anxiety, and depression [31], however Swedish massage is not recommended for FM patients [31]. Connective tissue in patients with chronic inflammation becomes dense [32]. Therefore, in the present study, we wanted to study the effect of moderate pressure massage on the dense connective tissue at the back of the neck and not Swedish massage. Ekici et al. [33] also support the application of connective tissue massage, among other manual therapies, and conclude that they improve pain, health status, and quality of life, so it could be used in the treatment of FM patients.

Furthermore, with the intention to improve knowledge about FM symptoms and its management, the aim of the study was to assess the effectiveness of a manual therapy technique performed with moderate digital pressure in FM patients on the variables of fatigue, pain, sleep, anxiety, and mood. This was done to provide another alternative treatment to pharmacological therapies for FM patients and to provide new evidence on the effects of manual therapy in FM patients.

## 2. Materials and Methods

### 2.1. Design

This was a randomised clinical trial in which the participants were allocated to one of two groups: an experimental group or a control group in order to determine the effectiveness of a manual therapy technique performed with moderate digital pressure on the variables of fatigue, pain, sleep, anxiety, and mood in women diagnosed with fibromyalgia. The subjects were randomly assigned electronically by block design into two arms (placebo and experimental group) using online computer software according to published recommendations [34]. A researcher who was not involved in the evaluations or interventions of this study prepared these envelopes.

### 2.2. Participants

Twenty-four female adult fibromyalgia syndrome patients aged 47 to 59 (53 ± 6) were recruited from local rheumatology practices in this randomised, controlled trial. The women studied had an average education level. They were recruited from public fibromyalgia associations in Alicante (Spain). All patients were chronic and they were interviewed and clinically evaluated in order to verify their eligibility by different physical therapists, using the same protocol and at the same time of day, both in the weeks of the intervention and in the week before and after the intervention (weeks 0 and 5). The participants of the manual therapy group (*n* = 14) were assisted with manual therapy sessions and those of the placebo group (*n* = 10) were treated with ultrasound sessions (US) performed without conductive gel and with the machine turned off as a placebo.

### 2.3. Declarations: Ethics Approval, Consent to Participate, and Consent for Publication

The present study was performed in accordance with the standards of the Helsinki declaration. The University Human Research Ethics Committee of Alicante University (Spain) granted approval to conduct a randomized trial (UA-2019-04-10) and all study participants provided written consent prior to the experiment. Furthermore, researchers kept confidential all participants’ personal data, codifying the personal information for that purpose. This study was also registered as a Clinical Trial in clinicaltrial.gov (Ref. NCT04158388).

### 2.4. Eligibility Criteria

Only female adults were included in this study. Before the study period, women who were assessed by a rheumatologist to determine the fibromyalgia diagnosis according to the criteria established by the American College of Rheumatology fibromyalgia diagnosis criteria in 2016 [3] were included in the study. In addition, the axial region of the neck and upper back must have been affected. Exclusion criteria from the study included: be performing simultaneously to this study some other physical therapy or physical exercise treatment; not having a sufficient cognitive level to collaborate in the study or not having the possibility of attending the established sessions.

### 2.5. Study Intervention

The intervention was performed in one month. Each group had two weekly sessions following the recommendations [26] for four weeks, with a total of 8 treatment sessions. The interventions of both groups were performed during the same time (15 min) and in the same anatomical area (posterior cervical musculature). Two massage therapy sessions were administered each week and were separated by at least 48 h. These patients were not receiving any other treatment at the time of the study. The evaluations and interventions of this study were conducted in the physiotherapy clinic. First, the women were assessed to determine if they met the eligibility criteria and data about their baseline characteristics were collected. Conversation between the participant and the massage therapist was kept to a minimum. The room was dimly lit and a sound machine was used to mask unwanted noises. Each session began with the subject draped with a sheet and in a prone position on a massage table while the therapist worked.

Recently, the Agency for Healthcare Research and Quality highlighted in its 2018 review update that chronic neck pain is one of the most common conditions in patients with chronic pain, the main characteristic of FM patients. Because Swedish massage is a massage that is characterised by general application, it is not recommended for FM patients [31].

For that reason, the manual therapy group received a manual therapy treatment based on a digital manual therapy. This manual therapy was performed on the connective tissue at the back of the neck with moderate pressure following bibliographic recommendations [29,35]; this is the right pressure to obtain the best benefits and this moderate pressure has to be reached in an increasing way [29]—that is, starting with light pressure until reaching the desired intensity. To ascertain moderate pressure, a numeric scale from 0 to 10 was used, where 6 was determined as the indicator of moderate pressure. The massage was performed for 15 min with the patient lying on their stomach and consisted of digital pressures, keeping the pressure at 6 on the number scale. This was done on the occipital and cervical musculature of the back of the neck, starting from the centre towards the periphery to increase blood circulation [32]. Participants were informed of the importance of not exceeding the intensity of 6 to avoid possible adverse and painful reactions, as suggested in other works [30]. The placebo group was treated with an ultrasound (US) (in off mode) without conductive gel. They were unaware at all times of the effect of the treatment they were receiving. All the interventions were performed by the same physiotherapist.

### 2.6. Outcome Measurements

Fatigue was measured using the fatigue severity scale (FSS); a Likert scale consisting of nine items that assess fatigue severity and functionality [36]. Items were rated on a scale of 1 to 7 according to their level of agreement with a given statement and included statements such as “I am easily fatigued” or “Fatigue interferes with carrying out certain duties and responsibilities”. Values for each item were averaged for a composite score, with higher scores indicating higher levels of impairment as a result of fatigue. FSS has been used in clinical practice for fatigue symptoms in people with chronic neck pain, showing high internal consistency (Cronbach’s alpha > 0.8) [37].

For pain perception, the visual analogical scale (VAS), commonly employed to assess the perception of somatic pain intensity, was used [38]. Validated in FM patients [39], this scale has a score from 0 to 10, where 0 refers to pain free or “no pain” and 10 refers to “worst possible pain”. It is a single-item scale [40]. Test–retest reliability has been shown to be good, but is higher among literate (*R* = 0.94, *p* < 0.001) than illiterate patients (*R* = 0.71, *p* < 0.001) before and after attending a rheumatology outpatient clinic [41].

Pittsburgh sleep quality index (PSQI) [42], validated in Spanish [43], assesses sleep quality. The PSQI consists of 19 survey questions related to sleep habits within the past month, including average sleep duration, sleep latency, and specific sleep-related problems such as reasons you have had trouble sleeping, administration of medications to help you sleep, and daytime sleepiness. These questions are grouped to form seven component scores, each with a range of 0–3. These component scores are then summed for a global sleep quality score (range 0–21), with higher scores reflecting worse sleep quality during the previous month. A total score greater than 5 indicates that the individual presents major dysfunctions in at least two components or moderate dysfunction in at least three components. The PSQI demonstrates good diagnostic sensitivity and specificity (44). It has internal consistency and a reliability coefficient (Cronbach’s alpha) of 0.83 for its seven components [44].

An abbreviated Spanish version of the profile of mood states (POMS-29) developed by McNair et al. [45] was used to assess the patients’ mood and mood changes. The POMS short form consists of 29 self-rated adjectives and each item of the POMS short form is scored on a 5-point Likert scale ranging from 0 (not at all) to 4 (extremely). It has been validated in the Spanish adult population [46]. The POMS measures: tension (reflecting an increase in musculoskeletal tension), anger (showing a mood of anger and dislike for others), vigour (representing a high-energy state), fatigue (representing a low-energy state), and depression (reflecting a low mood or a depressed mood). Total mood disturbance (TMD) was derived from POMS using the following formula, TMD = (sum of all subscales except vigour)—vigour. Internal consistency for the POMS-29 was reported to have a 0.76 to 0.95 Cronbach alpha rating [47].

### 2.7. Statistical Analysis

Statistical analysis of data was performed with JASP Team (2020) (JASP team, Amsterdam, The Netherlands; Version 0.12.2 computer software). For descriptive statistics (mean ± standard deviation) and inferential analysis, the Shapiro–Wilk test was performed to establish the normality distribution. Subsequently, independent sample t-tests were carried out to compare baseline values between groups. In addition, Levene’s test was conducted for equality of variances and analysis of covariance (ANCOVA) was applied (general linear model; 2 times × 2 groups; covariate: body mass index (BMI) to analyse the effects of the intervention on outcomes. Eta squared (η^2^) effect sizes for the time × group interaction effects were calculated. An effect of η^2^ ≥ 0.01 indicates a small, ≥0.059 a medium, and ≥0.138 a large effect. For those variables that showed significant main effects, post-hoc tests (Bonferroni) were performed. Furthermore, Pearson’s correlation test was used to establish relationships between the study variables. The level of significance was set at *p* ≤ 0.05. The effect size (ES) was calculated following the guidelines of Cohen [48]. The ES was considered negligible (<0.2), small (0.2–0.5), moderate (0.5–0.8), and large (>0.8).

## 3. Results

**Figure 1:**
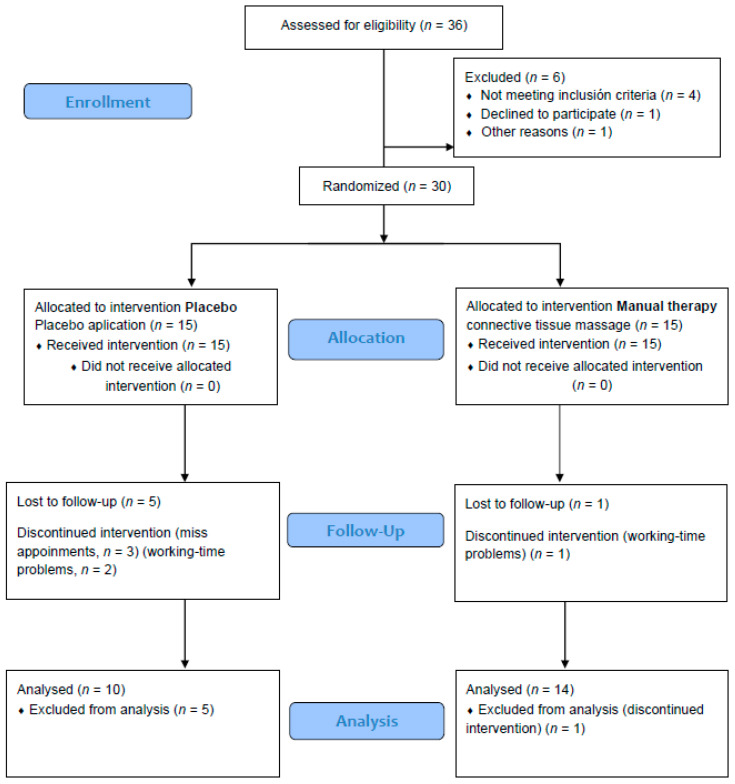
Consort 2010 flow diagram.

Thirty-six women were recruited for the present study and 36 were assessed for eligibility. Of these, four did not meet the inclusion criteria, one declined to participate, and another did not want to participate for other reasons, leaving 30 participants who were randomly allocated into the manual therapy group (MTG) and placebo group (PG). Over the follow-up period, six participants withdrew from the trial, one from the MTG and five from the PG. Therefore, 24 women were included in the analysis. Both groups of women did not differ on demographic variables. All withdrawals were due to personal reasons (Figure 1).

Table 1 shows descriptive statistics before and after the intervention, as well as the baseline comparison between groups (basal and post-intervention). It can be observed that the general sample presented homogeneous values at the initial assessment, with the exception of the fatigue variable (mean differences, MD: 0.9, *p* = 0.038).

**Figure 2:**
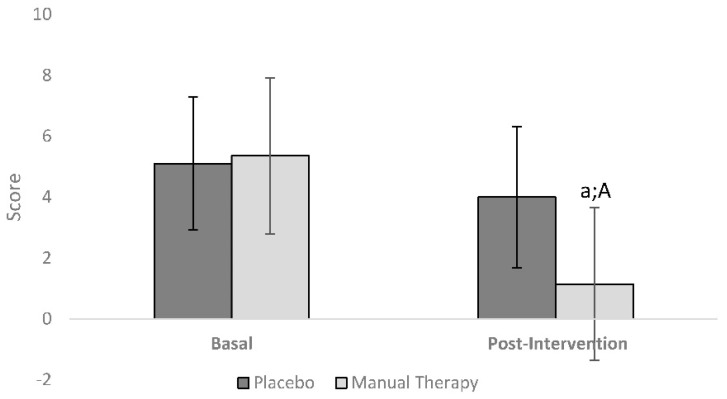
Effect of manual therapy. a: Differences between pre- and post-intervention (*p* < 0.001) for the pain scale; A: Differences between Placebo and Manual therapy groups (*p* = 0.044).

Table 2 presents the summary statistics of the ANCOVA analysis. The main analysis of the present study shows that there was a significant training × group difference (*p* < 0.001; η^2^ = 0.093) in the pain scale. The post hoc analysis showed a decrease between pre- and post-intervention in the manual therapy group (MD: 4.223, *p* < 0.001, ES: 2.072). In addition, inter-group differences were found (Figure 2) after the intervention (MD: 2.9, *p* = 0.044, ES: 0.593) in favour of the manual therapy group. However, no significant effects were found in any other variable.

The analysis of the correlations between all participants and each variable are shown in Table 3. A significant positive correlation was observed between fatigue and sleep (R = 0.411; *p* = 0.046). A significant positive association was also observed between the pain variable and the anger–hostility subscale of the POMS (R = 0.436; *p* = 0.033). Except the significant associations between the POMS questionnaire and its own subscales, significant correlations between variables were not identified.

## 4. Discussion

The aim of this study was to analyse the effectiveness of a manual therapy performed with moderate pressure on the variables of muscle fatigue, pain, sleep, and mood state in women with fibromyalgia. The effectiveness of a manual therapy in healthy people seems evident [35], however the literature shows little evidence of the effects of manual therapy in relation to the most characteristic symptoms suffered by FM patients [26]. Now, conclusions can be drawn on what the characteristics of manual therapy should be for FM patients—painless, progressive, and the intensity should gradually increase [26] from session to session depending on the patient’s symptoms. It has been observed [29] that the therapeutic benefit is greater than a direct deep application without any kind of light pre-pressure as a warm-up.

In terms of the benefits of manual therapy for patients, it promotes restful sleep, decreases anxiety and depression, and reduces the immediate and delayed perception of pain [25,26]. Regarding number of sessions, it is suggested to do at least 1–2 times per week [26], although the reason why this should be 2 and not a greater or lesser number of sessions is not clear.

It seems necessary to provide conclusive data to allow the use of manual therapy by health professionals as an alternative technique to other treatments with greater disadvantages such as drug treatments [49,50]. This research provides new insight into the use of manual therapy in FM; the treatment area. The manual therapy was carried out in the sensitive points of diagnosis, corresponding to the cervical area, where the patients experienced more pain. As in other investigations, a vibration manual therapy [51,52,53] was carried out with the fingertips, with moderate pressure [54] on the sensitive diagnostic points corresponding to the cervical area [55] for 15 min, twice a week for 4 weeks [26]. Moderate pressure is able to stimulate pressure receptors, which will lead to an increased vagal activity which seems to mediate the various benefits observed for manual therapy [56].

It is necessary to emphasise the importance of performing the massage with moderate pressure as described in the methodology and also to reach this pressure in an increasing way, as recommended by other authors [29]. The significant result of this study may be thought to be related to the pressure applied in the massage. Other studies where its application is not recommended, because of its moderately positive results, refer to the unpleasant pain that was experienced by the subjects due to the massage [57]. In the same direction as the results of our work, in the study carried out by Oliveira et al. [58], the effects of a massage therapy programme on cortisol concentration, pain intensity, quality of life, and perceived stress index of fibromyalgia patients were investigated. Subjects were treated with massage twice a week for three months. They suggest that the treatment improved quality of life, reduced the index of perceived stress, and reduced pain in these volunteers [58].

Based on the results of the FSS, the state of fatigue was not significantly reduced after the intervention. Fatigue is a factor that can be associated with morning stiffness [59]. The current limited number of treatment options for fatigue in FM patients has contributed to the increased level of disability in patients without apparent medical explanation [7]. In previous research, different methods have been used to measure muscle fatigue [9], for example, by examining static contractions of a single muscle in the upper or lower limb or by performing simultaneous contractions of several muscles. It has been observed that people with fibromyalgia have less muscle strength and voluntary resistance than sedentary controls [9]. This is why some people with fibromyalgia perceive a higher level of fatigue during activities of daily living (e.g., folding clothes, drying hair, or dressing). Therefore, it is suggested that the effect of moderate pressure manual therapy on the posterior cervical muscles does not seem helpful in improving the performance of household tasks due to the significant effect found for the reduction of fatigue.

One of the main reasons why FM patients seek medical care is musculoskeletal pain, along with sleep disorders [25]. As observed in the present study, after the intervention, pain decreased significantly based on the EVA scale. Other research [35] has also concluded that manual therapy is effective in improving health by reducing chronic back pain, migraines, and many other physical and psychological conditions in healthy patients. This clinical research is very helpful for understanding the benefits of manual therapy and accepting the technique as a treatment modality among health professionals [35]. More evidence is needed for FM patients to choose nonpharmacological treatments [60,61]. As previously observed, chronic neck pain is an unpleasant sensory experience that can have a negative psychological impact [62]. Patients with chronic neck pain experienced sleep deprivation, even when taking analgesics. Further, poor sleep quality is known to precede the onset of a depressed mood [63]. Therefore, it is suggested that if patients have experienced improvements in lower neck pain with massage therapy, they will also have experienced improvements in sleeping disorders.

Although manual therapy has been shown to promote restful sleep in FM patients [26], no significant results were obtained in the present study. Variables such as sleep and mood in the experimental group were positive but not significant. The systematic review by Choy [17] and others [16] suggests that exercise, cognitive behavioral therapy, and balneotherapy may improve sleep, but the data are low-quality evidence. Future research should determine the benefits of each of these treatments and evaluate their cost-effectiveness.

Persons with chronic pain are more likely to have depressive symptoms than those without pain [64]. FM patients who have a negative mood may have a poor perception of health [20]. This may be because psychosocial factors are known as risk factors for neck pain [65], and Blozik et al. [66] suggests depression and anxiety as major determinants of neck pain. Although this cannot be justified by the results obtained for mood state variables, future research will need to evaluate this adaptation because it is estimated that mood disorders are more than three times higher in FM subjects than in the general population [60].

Limitations of this study include the small sample size and the short-term assessment. In addition, while all attempts were made to standardize the delivery of the massage routine provided in this study, we did not control for the amount of pressure provided. This would be an important consideration for future studies, given that there is evidence that different amounts of pressure can elicit unique responses [35,53]. In other studies [35], the force applied by the therapist’s fingers was measured with ConTact type C500 sensors (Pressure Profile Systems, Los Angeles, CA, USA), which were pre-molded to fit the fingers of the massaging therapist and were fixed using latex cradles. The force data were collected with an imaging test, specifically electromyography. With this test, a digital pressure of 100 Hz was determined for the analysis. In addition, the scales of evaluation variables were self-reported measures, not objective measurements. It has not been possible to monitor directly with heart rate variability or muscle relaxation recording devices; therefore, this may have some influence on the final result. In addition, the impossibility of generalizing the results to the FM population should also be considered because the study included no men and the sample size is not a representation of the whole Spanish population with FM. Further, it has not been possible to control the duration of the disease and compare the efficacy of this type of therapy with other non-pharmacological ones. Finally, new lines of research are needed to shed more light on whether the benefits of manual therapy in FM patients could be sustained over time after treatment or whether they are only short-term benefits.

## 5. Conclusions

This investigation suggests that manual therapy with digital moderate pressure for 15 min on the posterior cervical musculature decreases the perception of pain in women with FM. In this sense, it seems that this technique could be considered as another alternative to pharmacological therapies for the treatment of FM.

## Figures and Tables

**Table 1:** Sample characteristics at baseline and post-intervention.

Variables	Placebo (*n* = 10)				Manual Therapy (*n* = 14)				Basal Differences		
	Basal		Post		Basal		Post				
	Mean	SD ^1^	Mean	SD	Mean	SD	Mean	SD	*t*	*p*	ES
Height (m)	1.6	0.1			1.5	0.1			0.768	0.451	0.318
Weight (kg)	70.7	11.0			68.2	11.7			0.528	0.603	0.219
BMI (kg/m^2^)	28.7	4.1			28.4	4.3			0.146	0.886	0.06
Fatigue (FSS)	4.7	0.8	5.3	0.7	3.8	1.0	4.6	0.7	2.213	0.038 *	0.916
Pain (VAS)	5.1	2.2	4.0	2.3	5.4	2.6	1.1	2.5	−0.257	0.799	−0.107
Sleep (Pittsburgh test)	11.3	2.7	10.3	2.7	9.6	4.1	7.9	3.1	1.159	0.259	0.48
POMS-29 total	138.2	13.0	128.1	10.5	131.4	17.3	120.9	16.3	1.042	0.309	0.432
Stress–Anxiety	10.9	4.4	8.3	3.5	11.0	5.2	6.0	2.7	−0.050	0.961	−0.021
Depression–Melancholy	6.0	2.5	4.0	3.6	6.3	4.7	4.2	4.4	−0.174	0.863	−0.072
Anger–Hostility	14.1	5.5	11.3	3.7	10.9	5.4	7.1	3.9	1.060	0.146	0.624
Vigor	6.3	3.0	7.4	4.1	8.4	5.5	7.3	5.4	−1.108	0.280	−0.459
Fatigue	13.5	2.8	11.7	4.5	11.8	4.3	9.1	4.1	1.105	0.281	0.458

^1^ SD = Standard Deviation; ES = Effect Size (d Cohen); BMI = Body Mass Index; FSS = Fatigue Severity Scale; VAS = Visual Analogical Scale; POMS-29 = Profile Of Mood States 29 items; * = mean differences were significant at *p* < 0.005.

**Table 2:** Effect of manual therapy (ANOVA).

Variable	Effect Time			Effect Time × Group		
	*F* ^1^	*p*	η^2^	*F*	*p*	η^2^
Fatigue (FSS)	0.899	0.354	0.013	0.352	0.559	0.005
Pain (VAS)	0.035	0.854	0.000	23.635	<0.001 *	0.093
Sleep (Pittsburgh test)	0.479	0.496	0.004	0.288	0.597	0.003
POMS total	1.187	0.288	0.009	0.020	0.890	0.000
Stress–Anxiety	0.005	0.945	0.000	1.580	0.222	0.023
Depression–Melancholy	0.224	0.641	0.001	0.007	0.936	0.000
Anger–Hostility	0.585	0.453	0.008	0.380	0.544	0.002
Vigor	2.902	0.103	0.017	2.269	0.147	0.013
Fatigue	1.256	0.275	0.008	0.380	0.544	0.002

^1^*F* = effect; n^2^ = eta squared (η^2^) effect sizes. In the model, all F were significant at *p* < 0.005. * = mean differences were significant at *p* < 0.005.

**Table 3:** Correlations between variables increments (post-intervention–basal).

Variables	Statistics	FSS ^1^ Fatigue	Pain	Sleep	POMS Total	Stress–Anxiety	Depression	Anger–Hostility	Vigor
Pain	Pearson (R)	−0.344							
	*p*	0.099							
Sleep	Pearson (R)	0.411 *	0.323						
	*p*	0.046	0.124						
POMS Total	Pearson (R)	0.073	0.253	−0.019					
	*p*	0.736	0.232	0.930					
Stress–Anxiety	Pearson (R)	0.007	0.114	−0.042	0.664 **				
	*p*	0.992	0.597	0.845	0.000				
Depression	Pearson (R)	0.106	−0.055	−0.015	0.794 **	0.415 *			
	*p*	0.623	0.799	0.945	0.000	0.044			
Anger–Hostility	Pearson (R)	0.091	0.436 *	0.062	0.795 **	0.590 **	0.406		
	*p*	0.672	0.033	0.773	0.000	0.002	0.049		
Vigor	Pearson (R)	−0.065	−0.157	0.122	−0.656 **	0.010	−0.585 **	−0.376	
	*p*	0.763	0.463	0.570	0.000	0.964	0.003	0.070	
Fatigue	Pearson (R)	−0.017	0.174	0.027	0.772 **	0.521 **	0.585 **	0.414 *	−0.427 *
	*p*	0.938	0.415	0.901	0.000	0.009	0.003	0.044	0.038

^1^ FSS = Fatigue Severity Scale; * *p* value < 0.05; ** *p* value < 0.01.
